# Single-Pulse Transcranial Magnetic Stimulation for the preventive treatment of difficult-to-treat migraine: a 12-month prospective analysis

**DOI:** 10.1186/s10194-022-01428-6

**Published:** 2022-06-06

**Authors:** J.O. Lloyd, B. Hill, M. Murphy, A. Al-Kaisy, A. P. Andreou, G. Lambru

**Affiliations:** 1 Headache Research-Wolfson CARD, Institute of Psychology, Psychiatry and Neuroscience, King’s College London, London, UK; 2 The Headache Centre, Guy’s and St Thomas NHS Foundation Trust, London, UK; 3 Institute of Psychology, Psychiatry and Neuroscience, King’s College London, London, UK

**Keywords:** Migraine, Chronic migraine, Refractory migraine, Transcranial magnetic stimulation, Neuromodulation, Non-invasive neuromodulation

## Abstract

**Background:**

Initial evidence have shown the short-term efficacy of sTMS in the acute and preventive treatment of migraine. It is unknown whether this treatment approach in the long-term is effective and well tolerated in difficult-to-treat migraine.

**Methods:**

This is a prospective, single centre, open-label, real-world analysis conducted in difficult-to-treat patients with high-frequency episodic migraine (HFEM) and chronic migraine (CM) with and without medication overuse headache (MOH), who were exposed to sTMS therapy. Patients responding to a three-month sTMS treatment, continued the treatment and were assessed again at month 12. The cut-off outcome for treatment continuation was reduction in the monthly moderate to severe headache days (MHD) of at least 30% (headache frequency responders) and/or a ≥ 4-point reduction in headache disability using the Headache Impact test-6 (HIT-6) (headache disability responders).

**Results:**

One hundred fifty-three patients were included in the analysis (F:M = 126:27, median age 43, IQR 32.3–56.8). At month 3, 93 out of 153 patients (60%) were responders to treatment. Compared to baseline, the median reduction in monthly headache days (MHD) for all patients at month 3 was 5.0 days, from 18.0 (IQR: 12.0–26.0) to 13.0 days (IQR: 5.75–24.0) (*P* = 0.002, *r* = − 0.29) and the median reduction in monthly migraine days (MMD) was 4.0 days, from 13.0 (IQR: 8.75–22.0) to 9.0 (IQR: 4.0–15.25) (*P* = 0.002, *r* = − 0.29). Sixty-nine out of 153 patients (45%) reported a sustained response to sTMS treatment at month 12. The percentage of patients with MOH was reduced from 52% (*N* = 79/153) at baseline to 19% (*N* = 29/153) at month 3, to 8% (*N* = 7/87) at month 12. There was an overall median 4-point reduction in HIT-6 score, from 66 (IQR: 64–69) at baseline to 62 at month 3 (IQR: 56–65) (*P* < 0.001, *r* = − 0.51). A total of 35 mild/moderate adverse events were reported by 23 patients (15%). One patient stopped sTMS treatment due to scalp sensitivity.

**Conclusions:**

This open label analysis suggests that sTMS may be an effective, well-tolerated treatment option for the long-term prevention of difficult-to-treat CM and HFEM.

## Introduction

Migraine is a common and often disabling neurological condition [1]. In patients with frequent migraine symptoms, pharmacological treatments constitute the main preventive strategy. However, the established migraine oral pharmacotherapy is often associated with efficacy, tolerability and adherence issues [2, 3]. Chronic migraine (CM), more than episodic migraine (EM) patients discontinue/switch between treatments largely because of lack of efficacy and/or tolerability issues [4]. Moreover, only a small proportion of CM patients adheres long term to pharmacological treatments over a period of 1 year [5]. Adherence and tolerability issues may be mitigated with the introduction of the novel monoclonal antibodies (Mabs) anti-calcitonin gene related peptide (CGRP), which seem to have a good tolerability profile in clinical trials, but less so in real-word analyses [6–8]. Furthermore, clinical trials data show a meaningful response rate of about 40–50% in CM and difficult-to-treat CM patients [9], highlighting the still unresolved unmet need in migraine management.

Non-invasive neuromodulation approaches have emerged as an alternative, or additional approaches to pharmacological treatments in headache disorders [10, 11] and other neuropathic pain conditions [12]. The rationale of these treatments is to improve the head pain and associated symptoms by altering the neural tissue activity of pathophysiologically relevant targets in a non-invasive fashion. One of the most promising of such treatments is portable, self-treatment single pulse transcranial magnetic stimulation (sTMS). In animal models of migraine, sTMS over the occipital cortex has been shown to interfere with mechanical and chemically induced cortical spreading depression (CSD) [13], which is considered the pathophysiological substrate of migraine aura [14]. Recent pre-clinical evidence suggested that this modulation of cortical activity in animal models may occur through interaction of sTMS with GABAergic circuits [15]. Additionally, sTMS may modulate spontaneous and C-fibre evoked trigeminovascular activity of third order thalamic neurons, suggesting a potential mechanism for migraine pain modulation [13]. In a sham-control clinical trial, sTMS administered over the occipital cortex has shown to be superior to sham as an abortive treatment in migraine with aura patients [16]. Open-label evidence testing the three-month efficacy of sTMS treatment in episodic migraine, suggested that almost half of patients obtained at least a 50% reduction in headache days [17]. Additionally, a company sponsored United Kingdom (UK)-based post market audit reviewed the effect of both acute and continuous use of sTMS device for 3 months in 449 patients with predominantly CM. Although the audit showed good short-term tolerability and effectiveness, the analysis was conducted only in 190 of the 449 patients (42%), suggesting caution in data interpretation [18]. In view of these evidence, Spring sTMS is CE-marked in Europe and obtained National Institute for Clinical Excellence (NICE) UK approval in 2014 [19] although without a technology appraisal guidance. Research in sTMS therapy lack independent, long-term effectiveness and safety data in CM patients who have already failed pharmacological approaches, which represents real world patients that mostly attend headache clinics. For this reason, we conducted a prospective clinical audit on the National Healthcare System (NHS) to evaluate the long-term effectiveness (1-year) safety and tolerability of sTMS in migraine, in line with the NICE UK audit framework [20]. The work constituted one of the Chapters of a PhD thesis of one of the contributing authors [21].

## Methods

This audit was part of a service evaluation of the non-invasive neuromodulation headache clinic of the Headache Service at Guy’s and St Thomas’ NHS Foundation Trust, London, UK, aiming at assessing the level of service and sTMS treatment effectiveness being provided by analysing completely anonymous data. New patients were included in the audit between January 2017 and May 2020. Audit under current national guidelines does not require research ethics committee review [22].

### Participants

Consecutive adult patients who received the sTMS treatment after attending the non-invasive neuromodulation headache clinic and meeting the International Headache Society (IHS) criteria for CM or for episodic migraine, with at least eight migraine days/month (high frequency) [23], who failed at least three established preventive treatments (beta-blockers, tricyclic antidepressants, antiepileptics, angiotensin II receptor antagonists), were included in the analysis. Patients who had previously failed to respond to botulinum toxin type A (BoNT/A) treatment were included in the audit. Treatment failure was defined as treatment discontinuation due to unacceptable side effects and/or absence of reduction in headache frequency, duration and/or severity after administration of a preventive medication at an adequate dose for at least 12 weeks. Contraindicated treatments were not considered as treatment failures. For patients who underwent a trial with BoNT/A, failure to obtain at least 30% reduction in headache days after two sets of injections 3 months apart was considered treatment failure as per NICE UK guidance [24]. Patients with medication overuse headache (MOH) were not excluded from the audit. When MOH was diagnosed as per IHS classification criteria [23], withdrawal attempts using outpatients pharmacological and non-pharmacological strategies were tried before entering the audit. Patients could continue oral preventive medications during treatment with sTMS, although we advised not to change the medications dose during the first three-month trial to avoid confounding the outcomes. Medications dose manipulation and/or introduction of new preventive treatments was allowed as per standard of care after month 3. Patients had to be compliant with device use, daily headache diary and 3-monthly headache impact test (HIT-6) completion to be part of this analysis. Patients with a personal history of epilepsy and/or implanted devices were excluded.

### Device use and treatment protocol

**Fig. 1:**
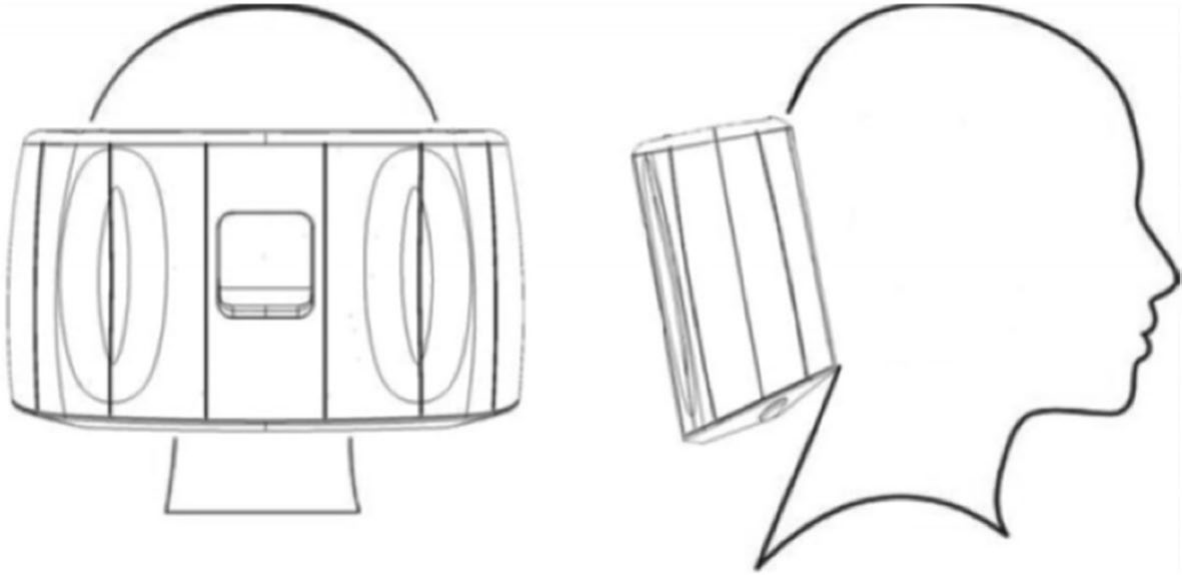
Spring TMS optimal placement and coil locations

Patients were demonstrated how to use the sTMS device by trained headache nurses (B.H., M.M.). Briefly, once turned on the sTMS device takes approximately 30–60 seconds for the capacitors to reach full charge, indicated by the LED progress bar surrounding the power button. Once at full charge the device can be positioned at the base of the skull (Fig. 1A) and a 0.9 T pulse with a time rise of 170 μs (measured 1 cm from the surface) can be delivered by pressing and holding the trigger buttons on either side of the device for at least 2 sec. As the treatment is delivered, the device produces an audible click, some patients have additionally described a tactile sensation. Pressing the power button recharges the device, for further treatments, otherwise the device shuts off after 10 seconds. The preventive treatment protocol consisted in delivering of two sequential pulses twice a day, to be titrated every 1–2 weeks to a maximum of six pulses three times daily, if needed (Table 1). Patients also had the option to use the sTMS as an abortive treatment. The abortive treatment protocol consisted in delivering as early as possible two sequential pulses every 15 minutes for 1–2 hours or until pain and symptoms resolve (Table 1). Patients were trialed on sTMS for a total of 3 months before establishing whether to continue the treatment or not.

**Table 1:** Single-pulse transcranial magnetic stimulation device treatment protocol

Preventive treatment titration protocol	
Week 1	Deliver 2 sequential pulses twice daily
Week 3	Deliver 2 sequential pulses three times daily
Week 5	Deliver 3 sequential pulses three times daily
Week 7	Deliver 4 sequential pulses three times daily
Week 9	Deliver 5 sequential pulses three times daily
Week 11	Deliver 6 sequential pulses three times daily
**Abortive treatment protocol**	Deliver as early as possible 2 sequential pulses every 15 minutes for 1–2 hours or until pain and symptoms resolve.

### Outcome measures

Details of the audit timeline are shown in Fig. 1B. A migraine-specific diary and the Headache Impact Test-6 (HIT-6) score were used to capture effectiveness and disability measures. Patients were required to produce a baseline headache diary and HIT-6 questionnaire for at least 1 month prior to treatment initiation, and to continue filling the headache diary on a daily basis along with monthly HIT-6 questionnaire following the conclusion of the 3-month trial. Data were entered in an electronic macro database for analysis.

The cut-off outcome for treatment continuation was reduction in the mean monthly headache days (MHD) of at least 30% after 3 months of treatment (headache frequency responders). This was derived by the 30% mean monthly headache days (MHD) reduction required by the NICE UK guidelines for continuation of other CM treatments under the NHS [25].

Furthermore, in a post-marketing UK audits, TMS therapy has shown to improve migraine-related impact using the HIT-6 score in a proportion of patients which was larger than the proportion of patients who experienced migraine frequency reduction at 12 weeks [18]. For this reason, we offered treatment continuation to patients who experienced at least a 4-point reduction in the HIT-6 (headache disability responders) even in the absence of a 30% MHD reduction. Other effectiveness outcomes analysed at month 3 included: changes from baseline in the MHD and in the monthly migraine days (MMD), proportion of patient with at least 50% and 100% reductions in MHD, change in monthly headache-free days, change in monthly abortive treatment intake use days and change in the proportion of patients with MOH. Headache frequency and disability responders at month 3 were formally re-assessed at month 12 to evaluate long-term efficacy outcomes of the therapy. Between month 3 and month 12 ad hoc visits were allowed.

A “headache day” was defined as a day with headache lasting for ≥4 hours and with a severity of ≥4/10 on a verbal rating scale (0, no head pain, 10 worst pain ever experienced). A “migraine day” was defined according to the IHS classification criteria [23], as a “headache day” with additional associated symptoms (nausea, vomiting, photophobia, phonophobia or motion sensitivity, or use of an abortive triptan. A “headache-free day” was defined as a day without any head pain. An “abortive treatment intake day” was considered any day where patients consumed oral or injectable abortive treatments for attempted headache relief. To assess whether any change in effectiveness measures was associated with improvement in headache-related disability, change in HIT-6 score was analysed. In view of the difficulty in collecting long-term reliable data on the abortive effect of sTMS in our patients, we focused our analysis on the preventive effect of this therapy, rather than the abortive effect.

Patients were asked about the development of adverse events (AEs) during the month 3 and month 12 (for responders only) assessments. Adverse events were graded as mild, moderate and severe.

### Statistical analysis

All outcomes pre- and post-sTMS treatment were measured on a continuous scale. For all measures considered here, data demonstrated a skewed distribution with a significant deviation from normal distribution (Kolmogorov-Smirnov test; *P* < 0.05). As a result, the Wilcoxon signed ranks test was used to compare the change in values over time. For independent group comparison the Mann-Whitney test was used. The Z value of these tests was used to calculate the effect size r, as Z statistic divided by the square root of the sample size (*N*).

*P*-values of less than 0.05 were regarded as evidence of a statistically significant result. Effects of sTMS between and within groups were analysed using SPSS statistics 23 (IBM, USA). Any missing values were treated in SPSS as discrete missing values. All data are provided as median (interquartile range (IQR), unless stated otherwise. Where relevant, patient numbers have additionally been given as a percentage of all registered patients.

## Results

### Demographic and baseline headache characteristics

**Fig. 2:**
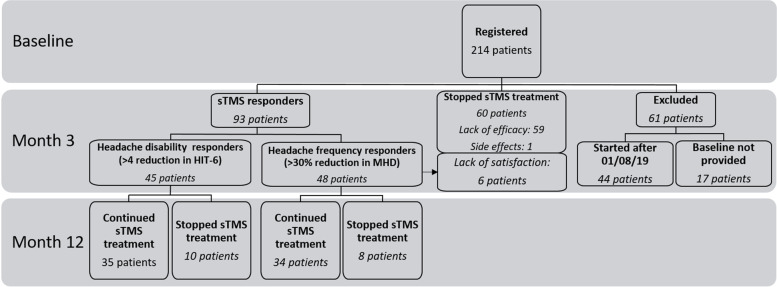
Audit flowchart

The sTMS service began in January 2017. Since then, a total of 214 patients have been prescribed the therapy (176 female; median age: 44.0 years IQR: 34.0–58.0). However, at the time of the analysis, 17 patients failed to provide completed headache diaries and HIT-6 at baseline and therefore were excluded from the audit analysis. Moreover, forty-four patients started treatment after 1st August 2019 and had to be excluded from the analysis due to lack of consistent data collection secondary to the ongoing COVID-19 pandemic (Fig. 2). A total of 153 patients completed the 3-month treatment trial and hence were included in the audit. All patients included in the analysis had completed headache diaries and HIT-6 at baseline and at least for the duration of the 3-month trial period. Demographic and clinical characteristics of the patients’ group at baseline are summarised in Table 2. All patients had failed at least three oral preventive treatments, of whom 45% (69/153) also failed BoNT/A before trialling sTMS. Most patients (93.0%) were classified in the severe impact category at baseline (HIT-6 score: 60–78).

**Table 2:** Demographic and clinical characteristics at baseline of all migraine patients (*N* = 153) treated with single pulse transcranial magnetic stimulation

	*All patients*	*CM Patients*	*HFEM patients*
Sex, M/F	27/126	27/101	0/25
Age (y), *Median (IQR)*	44.0 (34–58)	42 (33–55)	55 (43–64)
Diagnosis, CM/HFEM	128/25	128	25
Aura, *N* (%)	67 (44%)	56 (44%)	11 (46%)
Medication overuse, *N* (%)	68 (44%)	62 (48%)	6 (24%)
BoNT/A non-responders, *N* (%)	86 (56%)	80 (63%)	6 (24%)
Number of preventive treatments failed	5 (2–7)	4 (2–7)	3 (1–4.5)
CM duration (years), Median (95% CI)	13.5 (7.0 -, 18.0)	13.5 (7.0 -, 18.0)	N/A
Migraine days, *Median (IQR)*	13 (8.75–22)	15 (10–23)	8 (6–9.25)
Headache days, *Median (IQR)*	18 (12–26)	20 (15–29)	9 (8–10)
Headache free days, *Median (IQR)*	5 (0–13)	3 (0–11)	16.5 (14.25–19)
Abortive treatment intake days, *Median (IQR)*	9 (3.75–14)	9.5 (3–14)	8 (4.75–11.25)
HIT-6 score, *Median (IQR)*	66 (64–69)	66 (65–69)	64.5 (60.75–68)

*CM* Chronic migraine, *HFEM* High frequency episodic migraine, *BoNTA* Onabotulinum toxin A, *IQR* Interquartile range, *CM* Chronic migraine, *F* Female, *HFEM* High frequency episodic migraine; *HIT-6* Headache impact test-6, *M* Male, *N* Number, *y* Years

### Effectiveness outcomes at month 3 and treatment continuation

After three months exposure to sTMS therapy, 93 out of 153 patients (60%) were considered responders. Of these, 48 patients (31%) were “headache frequency responders” and obtained at least 30% reduction in MHD (median MHD at baseline: 16.5; (IQR: 10.25–21.0); median MHD at month 3: 5 (IQR: 3.0–9.0); (*P* < 0.001; *r* = − 0.87) and 45 patients (29%) were “headache disability responders” (median score at baseline: 66 (IQR: 65–68); median score at month 3: 62 (IQR: 58–65); (*P* < 0.001, *r* = − 0.67).

Compared to baseline, the median reduction in MHD for all patients at month 3 was 5.0 days, from 18.0 (IQR: 12.0–26.0) to 13.0 days (IQR: 5.75–24.0) (*P* = 0.002, *r* = − 0.29) and the median reduction in MMD was 4.0 days, from 13.0 (IQR: 8.75–22.0) to 9.0 (IQR: 4.0–15.25) (*P* = 0.002, *r* = − 0.29). At least a 50% reduction in MHD was obtained by 32 patients (21%) and three patients (2%) obtained a 100% reduction in MHD. There was an overall median 4-point reduction in HIT-6 score, from 66 (IQR: 64–69) at baseline to 62 at month 3 (IQR: 56–65) (*P* < 0.001, *r* = − 0.51). Treatment with sTMS led to a reduction in the percentage of patients with MOH, from 52% at baseline (*N* = 79/153), to 19% at month 3 (*N* = 29/153) (Table 3).

**Table 3:** Clinical characteristics of all patients using single-pulse transcranial magnetic stimulation (*N* = 153), headache frequency responders and (*N* = 48), headache disability responders (*N* = 45), and non-responders (*N* = 66) at baseline and Month 3

		***Headache days***	***Migraine days***	***Headache Free days***	***Abortive treatment-free days***	***HIT-6 Score***
***All Patients***	***Baseline***	18 (12–26)	13 (8.75–22)	5 (0–13)	9 (3.75–14)	66 (64–69)
	***Month 3***	*13* *5.75–24*	*9.0 (4–15.25*	*6.5 (0–16)*	*6 (1–11)*	*62 (56–65)*
	***Wilcoxon***	*P = 0.002*	*P = 0.002*	*P = 0.137*	*P < 0.001*	*P < 0.001*
***Headache Frequency Responders***	***Baseline***	*16.5 (10.3–21)*	*11 (7–14)*	*11 (4–15)*	*9 (4–14.75)*	*66 (63–68)*
	***Month 3***	*5 (3–9)*	*3.5 (2–8)*	*16 (11–24)*	*3 (1–9)*	*58 (53–63)*
	***Wilcoxon***	*P < 0.001*	*P < 0.001*	*P < 0.001*	*P < 0.001*	*P < 0.001*
***Headache Disability Responders***	***Baseline***	*20 (13–30)*	*14 (10–28)*	*2 (0–10.5)*	*10 (4.75–13)*	*66 (65–68)*
	***Month 3***	*23 (14–30)*	*12 (8–22)*	*1 (0–9.5)*	*8 (2–14)*	*62.5 (58–65)*
	***Wilcoxon***	*P = 0.925*	*P = 0.008*	*P = 0.922*	*P = 0.027*	*P < 0.001*
***Non-Responders***	***Baseline***	*18 (11–25)*	*14 (8–22)*	*5 (0–15)*	*9 (2.75–13)*	*67 (64–70)*
	***Month 3***	*16 (13–28)*	*13 (6.5–25)*	*3.5 (0–13)*	*8 (2.25–11.25)*	*66 (63–68)*
	***Wilcoxon***	*P = 0.413*	*P = 1.00*	*P = 0.801*	*P = 0.228*	*P = 0.009*

*HIT-6* Headache impact test-6

Fifty-nine patients (39%) were non-responders due to lack of effectiveness. One patient stopped the treatment due to an adverse event, namely scalp sensitivity. Of the 48 patients who achieved at least a 30% reduction in monthly headache days, six patients chose to discontinue sTMS due to lack of satisfaction with the treatment. Overall, a total of 87 patients (57%) continued to use the sTMS after the first 3-month trial.

### Effectiveness outcomes at month 12

**Fig. 3:**
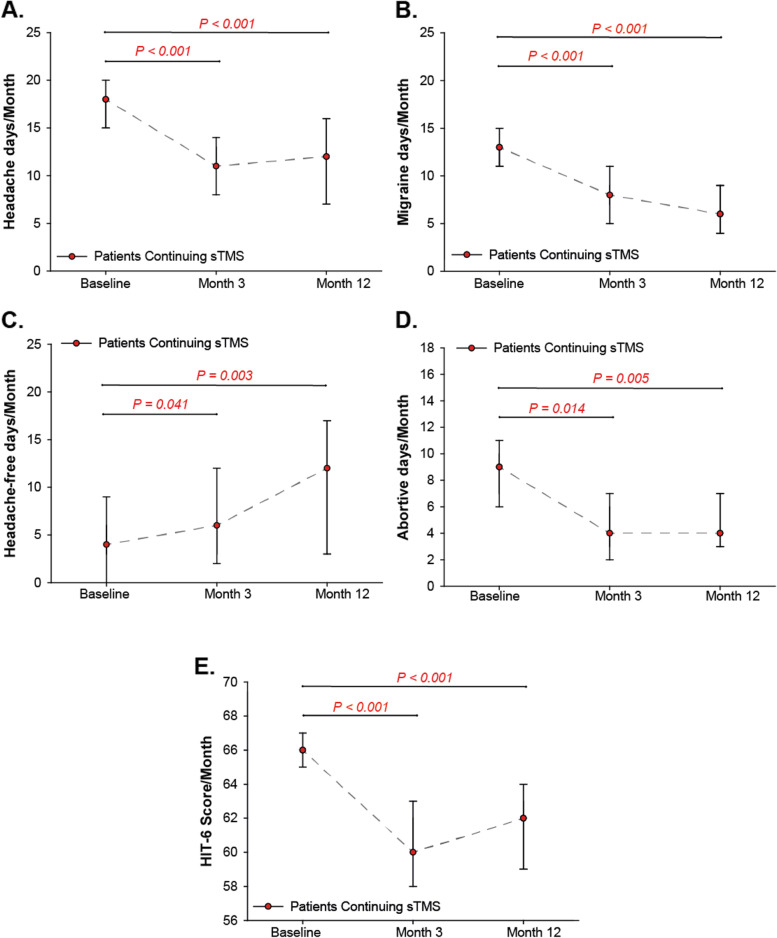
Effectiveness outcomes of patients continuing to use single-pulse transcranial magnetic stimulation at baseline and at 3 and 12 months

Of the total 153 patients, 69 patients (45%) remained responders and continued to use the sTMS treatment at month 12. This corresponded to 74% of responders at month 3. The monthly headache characteristics of patients who continued treatment for 12-months with sTMS (*N* = 69/153) are shown in Fig. 3. By month 12, further 18 patients discontinued the sTMS treatment due to lack of sustained effectiveness. In patients who continued the treatment at month 12 the reduction in MHD was sustained and remained significant compared to baseline (median MHD at month 12: 12.0, (IQR: 5.0–20.0), (*P* < 0.001, *r* = − 0.55). The reduction of MMD increased further compared to month 3 and remained significant compared to baseline (median MMD at month 12: 6.0 (IQR: 3.0–11.0), (*P* < 0.001, *r* = − 0.50). Treatment with sTMS significantly increased the number of headache-free days from 5 days (IQR: 0.0–13.0) at baseline, to 11.5 at month 12 (IQR: 0.0–22.0), (*P* = 0.003, *r* = − 0.39). The number of abortive treatment days was significantly reduced from 9.0 days at baseline, (IQR: 3.75–14.0) to 4.0 days at month 12 (IQR: 1.75–9.0), (*P* = 0.009, *r* = − 0.39). Continuation of treatment with sTMS reduced the percentage of patients with MOH from 52% (*N* = 79/153) at baseline to 19% (*N* = 29/153) at month 3, to 8% (*N* = 7/87) at month 12.

Responders at month 3 of sTMS therapy used a median of 12 pulses per day (IQR: 8–16), similarly to non-responders [median of 12 pulses per day (IQR: 8.25–18)]. Patients continuing sTMS at month 12 used a median of 15 pulses per day (IQR: 8–18). Of the patients who continued using sTMS for the full duration of 12 months, 25 patients (36%) were prescribed a median of two additional preventative treatments (IQR: 1–3). These included: including Onabotulinum toxin A, amitriptyline, nortriptyline, topiramate, propranolol, greater occipital nerve blocks, candesartan, gabapentin, pregabalin and pizotifen.

Compared to baseline, the reduction of median HIT-6 score in responders at month 12 was four points, from 66 (IQR: 64.0–69.0), to 62, (IQR: 56.25–65.0); (*P* < 0.001, *r* = − 0.51, Fig. 3E). The percentage of patients with severe headache-related disability was reduced from 93% at baseline to 63% at month 3 and 63% at month 12. Furthermore, 20% at month 3 and 24% of patients at month 12 reported some or little/ no headache impact compared to 4% at baseline (Table 4).

**Table 4:** Headache impact test-6 (HIT-6) headache disability categories at baseline and HIT-6 changes after daily single-pulse transcranial magnetic stimulation treatment for all patients at month 3 and for those who continued using the treatment at month 12

	Baseline (***N*** = 153) ***N*** (%)	Month 3 (***N*** = 93) ***N*** (%)	Month 12 (***N*** = 69) ***N*** (%)
**Severe impact (60–78)**	142 (93.0%)	59 (63.4%)	43 (63.0%)
**Substantial impact (56–59)**	4 (2.8%)	15 (16.1%)	9 (13.0%)
**Some impact (50–55)**	5 (3.5%)	15 (16.1%)	13 (18.5%)
**Little or no impact (< 48)**	1 (0.7%)	4 (4.3%)	4 (5.5%)

### Subgroup analysis

Further subgroups analysis looked at potential differences in the MHD at month 3 and month 12 between patients with migraine with aura and migraine without aura; patients who did not have BoNT/A and patients who had previously failed BoNT/A treatment; patients with and without MOH. The aura vs non-aura patients analysis showed no significant difference between month 3 (*N* = 68 vs 85) (*P* = 0.524, *r* = − 0.09) and month 12 (*N* = 38 vs 49) (*P* = 0.919, *r* = − 0.02). The change in MHD from baseline was not significantly different between CM and HFEM at month 3 (*N* = 61 vs 13) (*P* = 0.825, *r* = − 0.02) or month 12 (*N* = 43 vs 12) (*P* = 0.147, *r* = − 0.21). The pre-vs post-BoNT/A analysis also showed no significant differences between month 3 (*N* = 84 vs 69) (*P* = 0.139, *r* = − 0.21) and month 12 (*N* = 42 vs 45) (*P* = 0.665, *r* = − 0.08). Finally, no significant differences emerged from the comparison between patients with and without MOH: month 3 (*N* = 67 vs 86) (*P* = 0.637, *r* = − 0.07) and month 12 (*N* = 36 vs 51) (*P* = 0.082, *r* = − 0.36).

### Safety and tolerability

During the 3-month sTMS trial, a total of 35 adverse events were reported by 15% (*N* = 23/153) of patients. Four patients who did not experience adverse events during the first 3 months trial, reported side effects upon continuation of the therapy (18%, *N* = 27/153). Overall, the most frequent adverse events were: discomfort at the side of the sTMS delivery (34%, *N* = 12/35), worsening of the headache (28%, *N* = 10/35), and nausea (11%, *N* = 4/35). Adverse events were transient, lasting for seconds to minutes following sTMS stimulation and described as mild or moderate in the vast majority of patients. One patient stopped sTMS treatment due to scalp sensitivity.

## Discussion

This is the first independent, large, prospective analysis evaluating the effectiveness and tolerability of sTMS in difficult-to-treat CM or HFEM with and without aura patients, with and without MOH. This is the first long-term analysis of non-invasive neuromodulation treatments ever conducted in headache disorders. sTMS treatment seems effective in over half of our patients and very well tolerated, with infrequent and often mild side effects and a very low percentage of patients discontinuing the treatment due to side effects. Effectiveness was sustained over time, resulting in long-term meaningful improvement of migraine symptoms in 45% of patients, regardless of MOH and of the level of refractoriness. Interestingly, the majority of responders experienced a substantial reduction in headache/migraine days (> 50%) and some patients became completely migraine-free throughout the follow-up period.

These findings are clinically relevant in view of the type of patients treated. Initial evidence suggests that CM and HFEM may be the same condition and may be better grouped together given their response to treatment and level of disability [26]. Indeed, both CM and HFEM are generally challenging conditions to treat. Furthermore the vast majority of patients included would meet the recently updated EHF criteria for resistant migraine since they failed at least three drug classes with evidence in migraine prevention [27]. For such patients the treatment options are currently limited and often lead to disappointing results, leaving them with severe headache-related disability, as reported by our patients at baseline. Current treatment options for resistant CM include Onabotulinum toxin A (BoNTA), which is a very effective treatment [24]. However, about 40% of patients do not report a satisfactory response. Moreover, BoNTA has been shown to be more expensive than sTMS on the UK NHS and with regular three-monthly administration regimen, may put long-term unsustainable pressure on headache clinics due long-term capacity issues, leaving some patients without treatment continuity [28]. For BoNTA non-responders who have also failed three classes of medications, evidence-based treatments with sustained long term follow-up data are very limited [24]. The CGRP monoclonal antibodies response rate in CM varies between 27 and 41% [29–32]. Erenumab has been recently shown to be a potentially effective treatment in refractory CM, though long-term data are missing [8]. Invasive neuromodulation, namely occipital nerve stimulation (ONS) holds a large body of long-term open label evidence [33, 34]. However, the treatment’s tolerability and costs limit its widespread use. Furthermore, when properly phenotyped refractory CM patients were evaluated, the long-term beneficial response rate of ONS in open-label studies was obtained by about a third of the patients [34]. On the other hand, other non-invasive neuromodulation treatments for migraine, like vagus nerve stimulation were shown to be ineffective in refractory migraine patients [11]. Often real-world data is produced on difficult-to-treat patients assessed in tertiary headache clinics. NICE UK recommend sTMS for patients who have failed three or more preventative treatments. However, given its extremely good tolerability and cost profile, at least on the NHS, this therapy may be offered in patients earlier on in the preventive treatment pathway.

One of the important roles of real-world analysis is to produce data on long-term sustainability of treatments. BoNTA showed reasonably good long-term data with only a small proportion of patients discontinuing the treatment at 12 and 24 months [24]. An open-label extension of pivotal topiramate trials showed discontinuation rates during the open-label extension phase of 8.6% for those patients who had already received topiramate [35]. Out of 383 patients followed-up in a 5-year extension of a randomised clinical trial of erenumab in the prevention of episodic migraine, 34% of patients (*N* = 132) discontinued erenumab [36]. In our group of patients, only a percentage of 16% discontinued the treatment. This reflects a promising sustained effectiveness of sTMS, given the chronic subtype and the resistant nature of the condition our patients had.

Although changes in migraine or headache days have been recommended overtime as pivotal outcomes in migraine clinical trials [37], patients reported outcomes, namely quality of life measures and migraine-related disability scales, are becoming key in appreciating the effect of a treatment in patients’ day-to-day life and in justifying treatment continuation [38–40]. It is unclear how many points reduction on the HIT-6 scale are clinically relevant [41], especially in the difficult-to-treat migraine population, though reduction between 2.5 and six points seem to largely reflect meaningful effectiveness [42]. Our cut-off of four points reduction on the HIT-6 scale was based upon the refractoriness to treatment of our group of patients. The use of the HIT-6 changes to evaluate treatment continuation, alongside the traditional effectiveness outcomes (headache and migraine frequency changes) highlighted a substantial proportion of patients with < 30% improvement in MHD but with meaningful reduction of the HIT-6 score, who wanted to continue the treatment for 12 months, implying a positive effect of sTMS to their symptoms. Future research should focus on evaluation of migraine treatments-related outcomes and healthcare policy decision makers may need to consider introduction of headache-related disability scales as a valid treatment outcome to justify treatment continuation.

The main limitation of this audit is the open label design, hence the lack of a control group. In CM preventive treatment trials, a significant placebo response is often noted [43, 44]. The more recent clinical trials assessing the efficacy of the anti-CGRP monoclonal antibodies reported a placebo response ranging between 15% and 39% [45–47]. Interestingly, the studies testing the effect of the anti-CGRP Mabs in treatment resistant migraine patients, showed that the higher the number of preventive treatments were failed, the lower the placebo effect was, suggesting that in the difficult-to-treat CM population, the placebo effect may not interfere significantly with the biological effect of an active treatment. In our analysis, a placebo effect may have inflated the response rate during the first 3 months trial, especially within the “headache disability responders”. However, a good proportion of them discontinued the treatment between month 4 and month 12 and the proportion of long-term responders was reduced to 45%. This corresponds to a 16% reduction in percentage of responders, which is similar to the 14% placebo response found in a refractory CM study [47]. It is therefore unlikely that the long-term symptoms improvement could be explained by a placebo effect alone. A proportion of patients introduced oral preventive treatments after 3 months exposure to sTMS. This may constitute a confounding factor to the overall long-term effectiveness of sTMS. However, given the rarity of studies assessing long-term effect of treatments in resistant migraine, it is difficult to establish if this percentage of patients on polytherapy would be normally seen in clinical practice with any preventive treatments used in this population.

The strengths of this report include the refractoriness of the group of patients treated, which reflects the type of complex and difficult-to-treat patients seen in tertiary headache clinics; the long follow-up, which was a missing information in the non-invasive neuromodulation headache literature and provides pivotal clinical information on the real utility of this treatment approach.

In conclusion, sTMS therapy appears safe and well tolerated and effective treatment for the prevention of migraine in a meaningful proportion of treatment resistant CM/HFEM patients with and without MOH. sTMS’ s beneficial effect consisted in reduction in traditional headache effectiveness measures, namely monthly headache and migraine frequency, but also in a remarkable improvement of headache-related quality of life. The improvement was sustained overtime in a significant proportion of patients and not influenced by the level of patients’ refractoriness. Given its favourable cost and safety profiles, sTMS may be positioned before more expensive treatments in the migraine treatment pathway.

## Data Availability

Anonymized data are available from the corresponding author upon reasonable request.

## Abbreviations

AEs: Adverse events
BoNT/A: Botulinum toxin type A
CGRP: Calcitonin gene-related peptide
CM: Chronic migraine
EHF: European Headache Federation
HIT-6: Headache Impact Test, 6th Edition
ICHD: International Classification of Headache Disorders
IHS: International Headache Society
IQR: Interquartile range
MABs: Monoclonal antibodies
MHD: Monthly headache days
MMD: Monthly migraine days
MOH: Medication overuse headache
NHS: National Health System
NICE: National Institute for Health and Care Excellence
sTMS: Single pulse transcranial magnetic stimulation
U.K.: United Kingdom
